# The impact of environmental and climatic variation on the spatiotemporal trends of hospitalized pediatric diarrhea in Ho Chi Minh City, Vietnam

**DOI:** 10.1016/j.healthplace.2015.08.001

**Published:** 2015-09

**Authors:** Corinne N. Thompson, Jonathan L. Zelner, Tran Do Hoang Nhu, My VT Phan, Phuc Hoang Le, Hung Nguyen Thanh, Duong Vu Thuy, Ngoc Minh Nguyen, Tuan Ha Manh, Tu Van Hoang Minh, Vi Lu Lan, Chau Nguyen Van Vinh, Hien Tran Tinh, Emmiliese von Clemm, Harry Storch, Guy Thwaites, Bryan T. Grenfell, Stephen Baker

**Affiliations:** a Oxford University Clinical Research Unit, Wellcome Trust Major Overseas Programme, Ho Chi Minh City, Vietnam; b Centre for Tropical Medicine, Nuffield Department of Medicine, University of Oxford, United Kingdom; c The London School of Hygiene & Tropical Medicine, United Kingdom; d Robert Wood Johnson Foundation Health and Society Scholars Program, 701A Knox Hall, Columbia University, New York, NY 10027, USA; e Wellcome Trust Sanger Institute, Hinxton, Cambridgeshire, United Kingdom; f Children's Hospital 1, Ho Chi Minh City, Vietnam; g Children's Hospital 2, Ho Chi Minh City, Vietnam; h Hospital for Tropical Diseases, Ho Chi Minh City, Vietnam; i Department of Ecology and Evolutionary Biology, Princeton University, NJ, USA; j Brandenburg University of Technology, Cottbus, Germany; k RAPIDD Program, Fogarty International Center, National Institutes of Health, Bethesda, MD, USA

**Keywords:** Diarrhea, Climate change, Environment, Spatial risk, Mixed effects

## Abstract

It is predicted that the integration of climate-based early warning systems into existing action plans will facilitate the timely provision of interventions to diarrheal disease epidemics in resource-poor settings. Diarrhea remains a considerable public health problem in Ho Chi Minh City (HCMC), Vietnam and we aimed to quantify variation in the impact of environmental conditions on diarrheal disease risk across the city. Using all inpatient diarrheal admissions data from three large hospitals within HCMC, we developed a mixed effects regression model to differentiate district-level variation in risk due to environmental conditions from the overarching seasonality of diarrheal disease hospitalization in HCMC. We identified considerable spatial heterogeneity in the risk of all-cause diarrhea across districts of HCMC with low elevation and differential responses to flooding, air temperature, and humidity driving further spatial heterogeneity in diarrheal disease risk. The incorporation of these results into predictive forecasting algorithms will provide a powerful resource to aid diarrheal disease prevention and control practices in HCMC and other similar settings.

## Highlights

• Mixed effect modeling of hospitalized diarrheal admissions.
• Diarrhea is seasonal and spatially heterogeneous in Ho Chi Minh City, Vietnam.
• Low elevation is a significant risk for pediatric diarrheal disease.
• Spatial heterogeneity in risk is due to flooding and other environmental factors.
• Climate based predictive modeling may help reduce diarrheal epidemics in the future.

## 1 Introduction

Diarrheal disease is a leading cause of childhood morbidity and mortality worldwide, with an estimated 1.7 billion infections and 0.7 million deaths annually (Walker et al., 2013). Oral rehydration, antimicrobials, and intravenous fluids are proven to save lives in outbreaks, and prevention measures such as vaccination and improved water, sanitation and hygiene (WASH) reduce endemic diarrheal disease incidence (Bhutta et al., 2013, Santosham et al., 2010). Nonetheless, the general uptake and sustainability of these measures globally is limited (Bhutta et al., 2013, Forsberg et al., 2007). Innovative techniques to address the massive burden of diarrheal disease are required. Akanda and colleagues recently proposed that climate-based surveillance should be factored into control strategies for diarrheal disease (Akanda et al., 2014). The authors argued that using climatic and remote sensing data can help predict diarrheal epidemics driven by environmental exposures in vulnerable communities up to three months prior to occurrence. This early warning mechanism allows time to preemptively administer an immunization program or provide WASH interventions such as water filtration devices (Akanda et al., 2014, Akanda et al., 2012). Understanding the nature and magnitude of the relationships between diarrheal disease epidemiology and factors such as temperature, flooding and humidity should allow for the development of setting- or regionally-specific predictive models for climate-based early warning systems (Jutla et al., 2013).

We sought to describe the impact of environmental and climate conditions on the risk of diarrheal disease in Ho Chi Minh City (HCMC) in southern Vietnam. Considered an “Asian coastal megacity”, HCMC is a rapidly industrializing low-lying city of 7.5 million people (The World Bank, 2010). Diarrheal disease remains a problem in Vietnam, with an estimated incidence of 271/1000 infant years in children under 12 months of age (Anders et al., 2015). Management of diarrhea in this resource-limited setting has become more challenging in recent years because of diminishing antimicrobial susceptibility in causative bacteria (Le et al., 2009, Vinh et al., 2009). Although seasonality of hospitalized diarrheal disease in this area has previously been recognized (Thompson et al., 2015), the effects of climate and weather on reported diarrhea risk have not been quantified.

In 2012 the UN reported that 98% and 93% of urban Vietnamese have access to improved drinking water sources and sanitation facilities, respectively (World Health Organization and UNICEF, 2014). However, rapid population growth, lack of investment and aging water and sewage infrastructure have led to alarmingly polluted waterways and expose residents of HCMC to considerable risk (Ha et al., 2008, Vo, 2007, Wust et al., 2002). The water of the Saigon and Dong Nai rivers is used as the drinking water for the population of HCMC, with limited purification or controls on industrial and domestic waste discharge (Hong et al., 2000, Vo, 2007). For example, it is estimated that only 40% of waste water from industrial and hospital sources discharged into the Saigon River is treated (Vo, 2007). Furthermore, poor residents who live along the canals and in settlements in HCMC often resort to groundwater use, which has been shown to be contaminated with human and animal excreta as well as toxic industrial waste (Ha et al., 2008, Hong et al., 2000).

Characterizing the role of environmental factors in diarrheal disease risk is challenging due to a general lack of pathogen-specific diagnoses, with “all-cause” diarrhea data reflecting a combination of viral, parasitic and bacterial pathogens, which vary in transmission dynamics and sensitivities to environmental conditions. Furthermore, seasonal patterns of diarrheal disease hospitalization may correlate with seasonal environmental factors even when no causal relationship exists; i.e. the transmissibility of the underlying pathogens may vary seasonally as a function of seasonal variation in contact behavior (Nichols et al., 2012, Pitzer et al., 2009), birthrates and other demographic factors (Cannon et al., 2009), or other unmeasured weather and climate factors (Kolstad and Johansson, 2011, Levy et al., 2009, Wu et al., 2013). To quantify the impact of weather and the environment on diarrheal risk in HCMC, we used a large, spatially explicit dataset of pediatric diarrheal admissions from three hospitals. We explored inherent seasonality of reported diarrhea at a city level and examined the sensitivity of each district to the effects of climate on risk to highlight localized drivers of diarrhea. Our work identifies marked spatial heterogeneity in risk of reported diarrhea in HCMC corresponding to differences in elevation and localized sensitivity to seasonal climate patterns; such results can be used to inform future climate-based predictive algorithms in this region for efficient targeting of public health and economic resources.

## 2 Methods

### 2.1 Patient data

To investigate the impact of local environmental factors on rates of diarrheal disease hospitalization across HCMC, data on all children <16 years of age, who were living in HCMC and admitted with diarrheal disease was collected from three large hospitals in HCMC for the period 2005–2010, inclusive. Data from Children's Hospital 1 (CH1) and Children's Hospital 2 (CH2) spanned the period from 2005 to 2010. Data from 2008 to 2010 was collected from the Hospital for Tropical Disease (HTD). For each patient, information on age, sex, date of admission, ward and district of residence, and ICD10 code on admission were provided. Any patient with an ICD10 diagnosis contained under the WHO-defined heading of “Intestinal infectious diseases” (codes A00-A09) (WHO, 2010) was classified as having diarrheal disease. A total of 443,295 children resident in HCMC under the age of 16 were admitted to CH1 and CH2 over the six-year period, of which 51,599 (11.6%) were admitted with diarrheal disease. HTD reported a total of 36,624 admissions from 2008 to 2010, of which 7174 (19.6%) were admitted with diarrheal disease.

### 2.2 Environmental and demographic data

**Fig. S1:**
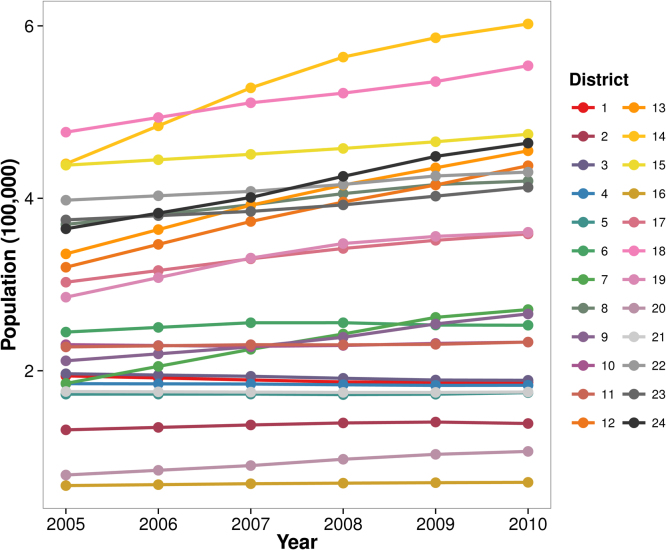


Weather and climate data measured at the city level were obtained from the Ministry of Natural Resources and Environment of Vietnam (Loc, 2013). These include: citywide weekly average relative humidity (%), temperature (°C), rainfall (mm) and level of the Don Dien River of HCMC (cm above or below long-term average river level) during 2005–2010. To assess the relative impacts of seasonal variation in these environmental drivers, these covariates were standardized to have zero-mean and unit variance. The minimum and maximum values of the mean-standardized climate factors are as follows, (1) humidity: −2.7, 2.2; (2) temperature: −3.2, 3.1; (3) rainfall: −0.9, 4.5; (4) river level: −2.0, 2.3. Shuttle Radar Topography Mission (SRTM) elevation data was obtained from the CGIAR Consortium for Spatial Information (CGIAR-CSI) (Jarvis et al., 2008). Data on ward-specific populations were extracted from the 2009 Population and Housing Census (General Statistics Office of Vietnam, 2010). District-level populations were available at the beginning of 2005 and for each year from 2008 to 2010 from the 2011 Statistical Yearbook of HCMC (Statistical Office in Ho Chi Minh City, 2012). Weekly population sizes for each district were estimated from annual census estimates by linear interpolation (see Fig. S1).

### 2.3 Spatial information

There are 24 administrative districts in Ho Chi Minh City (HCMC) (Table S1) with a median area of 22 km^2^, range (4–704 km^2^) and median population density of 20,340/km^2^ (range: 98–45,370/km^2^). Additionally, within each district there are a series of wards that vary in size and number. There are a total of 322 wards within HCMC, with a median area of 1.2 km^2^ (range: 0.1–122 km^2^), and median population density 24,200/km^2^ (range: 47–125,000/km^2^).

Home addresses for all diarrheal disease inpatients were geocoded to district and ward centroids of HCMC. Spatially smoothed rates of hospitalization at the ward level were estimated using Empirical Bayesian Kriging (EBK) (Pfeiffer et al., 2008). EBK allows estimation of spatially smoothed small-area rates by combining local information with weighted data from neighboring areas. Reported rate surface predictions and their associated variance (reflecting uncertainty in variogram estimation) were generated for the entirety of HCMC. More specifically, a new semivariogram is estimated for each of a set of overlapping subsets of the input data. These are then combined to obtain smoothed rates while accounting for variability in the strength of local spatial correlation across the map. EBK and mapping were performed using ArcGIS v10.2 (ESRI, California).

### 2.4 Multivariate model

We utilized a Poisson generalized linear mixed model (Gelman and Hill, 2006) to distinguish variation in reported diarrheal disease risk from seasonal epidemics and other city-level seasonal factors from district-level variation in response to climate factors. This model, with the outcome of monthly rate of diarrheal cases per population per district, included: (1) fixed-effects for district attributes (e.g. elevation), (2) monthly random intercepts to account for seasonal and district-level heterogeneity, and (3) district-level random intercepts and slopes to account for variation in response to city-level weather and climate factors. To ensure that our parameter estimates reflect per-capita rates, all models included an offset term for the logarithm of the population of district i at week t. Mixed effects analysis was performed in R (v3.02) using lme4 (v1.1–7) for R (Bates et al., 2014); plots were created using ggplot2 (v0.93) (Wickham, 2009). Goodness-of-fit analyses were performed and are described in the SI.

We included a fixed effect term to adjust for distance to the largest and most centrally located hospital (CH1) to ensure our estimates of district-level effects were not artifacts of differential reporting due to travel distance. All three hospitals are located in central HCMC, and inclusion of terms for distance to all hospitals instead of CH1 alone did not impact our results. We included lag terms for the district-level per-capita diarrheal hospitalization rate over the preceding eight weeks to account for temporal autocorrelation. Next, risks experienced simultaneously across all districts of HCMC were represented by random intercepts for each month from 2005 to 2010. The use of a monthly interval for these effects ensured that if a temporally varying district-level weather or climate effect was strongly correlated with citywide seasonal patterns, these district-level effects were still captured in the weekly data. In addition, the use of a monthly random intercept allowed us to capture short-term spatial lags in these effects.

**Fig. S2:**
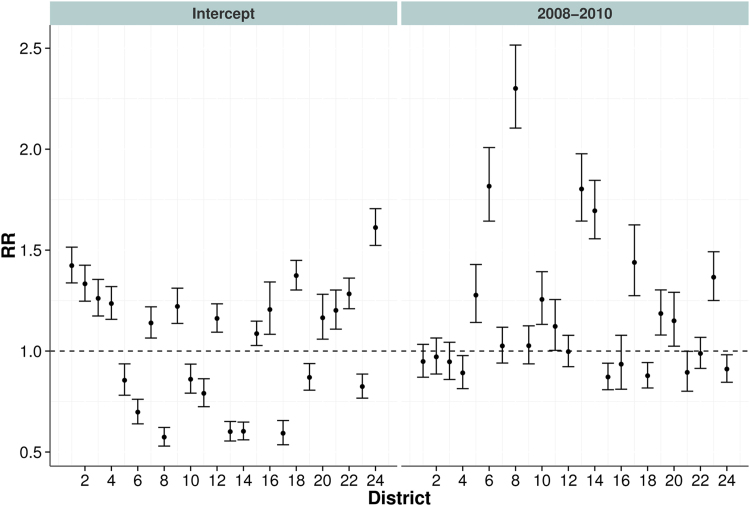


Next, we included a random intercept for each district to account for baseline variation in district-level diarrheal hospitalization. We also included district-level random slope terms for standardized values of city-level climate variables. Changes beginning around 2008 to policies regarding health insurance for children under the age of six years may have impacted reporting in some locations around this time (specifically the catchment areas of CH1 and CH2) (Shieh et al., 2013). To account for changes in reporting following this policy shift, a district-level random slope term indicating which weeks occurred during and after 2008 was included in the model in addition to the district-level weather and climate random effects (Fig. S2). Population density was not associated with variation in district-level risk and was not included in the final model.

To assess the agreement between our model and the district-level data, we generated 1000 simulated datasets and visually compared the range of these simulated values to the weekly data. We also calculated deviance residuals to provide a quantitative measure of goodness-of-fit. The resulting district-level predictions (Fig. S5) and deviance (Fig. S6) displayed a generally good agreement between the fitted model and the source data. To ensure that district-level autocorrelation was adequately accounted for, we examined the partial autocorrelation function of the deviance residuals for each district up to a 10-week lag (Fig. S7). The partial autocorrelation function (PACF) represents the autocorrelation between two points t and t+1 in a series after adjustment for the previous *t*−1 terms, suggests that district-level temporal autocorrelation is adequately accounted for by our model. The resulting figures indicate good agreement between the model and data at the district level.

Ethical approval for this study was granted by all local hospital ethical committees and the Oxford Tropical Research Ethics Committee.

## 3 Results

### 3.1 Patterns of diarrheal disease reporting in Ho Chi Minh City

Of the 479,919 childhood admissions across three hospitals over 2005–2010, 58,773 (12.3%) were attributed to diarrheal disease. Only ICD10s of the acute respiratory infection syndrome (J00-J06) (WHO, 2010) accounted for more admissions (14%). The most commonly documented diarrheal ICD10 codes were A09 (Gastroenteritis [GE]/colitis of infectious origin):27%, A09.1 (diarrhea and GE of presumed infectious origin with mild dehydration):27%, A09.2 (diarrhea and GE of presumed infectious origin with severe dehydration):19% and A04.9 (bacterial intestinal infection):9%.

The median age of the hospitalized diarrheal cases in HCMC was 1.2 years (interquartile range: 0.7–2.1 years), and 63% of these cases were male.

**Fig. S3:**
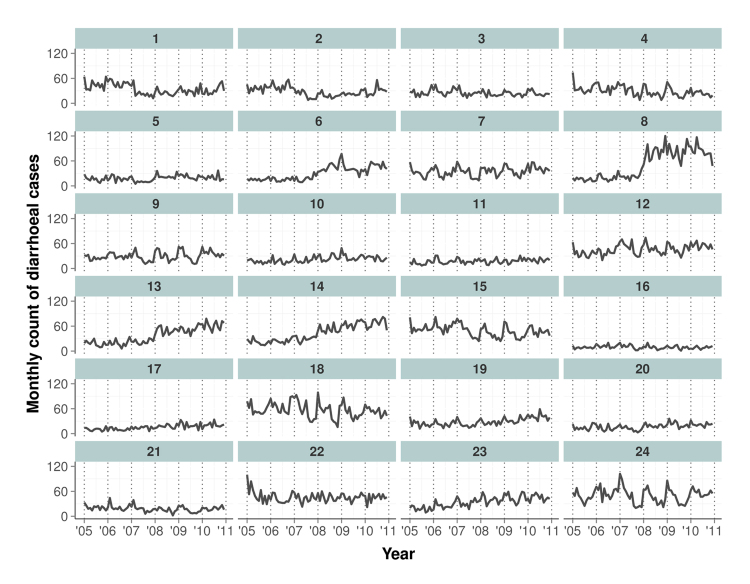


**Fig. 1:**
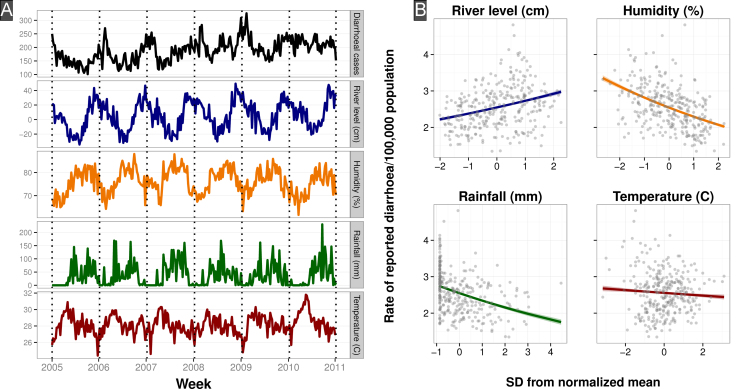
Time series and correlation of diarrheal case counts and climate factors in Ho Chi Minh City. (A) From top to bottom: Individual weekly time series (period 2005–2010) of total citywide reported cases of diarrhea recorded at the three study sites, average river level of the Don Dien river in southern HCMC in cm, average weekly relative percent humidity, average weekly rainfall in cm and the average weekly temperature in Celsius. (B) Scatterplots of weekly diarrheal case counts and normalized average weekly river level and citywide humidity, rainfall and temperature. The climate variables have been normalized to zero mean and unit variance. The colored lines represent the fitted Poisson model. (For interpretation of the references to color in this figure legend,the reader is referred to the web version of this article.)

A consistent pattern of seasonality in diarrhea hospitalizations was apparent from the citywide count data (Fig 1A). Throughout the sampled years the burden of diarrheal disease was highest during the drier months from January to March with a mean of 10% of yearly cases reported in each of these months (range: 7.5–12.4%). There was a corresponding trough in August–September, with a mean of 7% of annual cases reported during each month (range: 6.1–8.2%). Individual district-level time series varied in magnitude and variability of the yearly peak of reported cases in the dry season (Fig. S3). The overall rate of reported diarrhea across HCMC did not differ spatially when stratified by age of patients or by season (dry and wet).

**Fig. 2:**
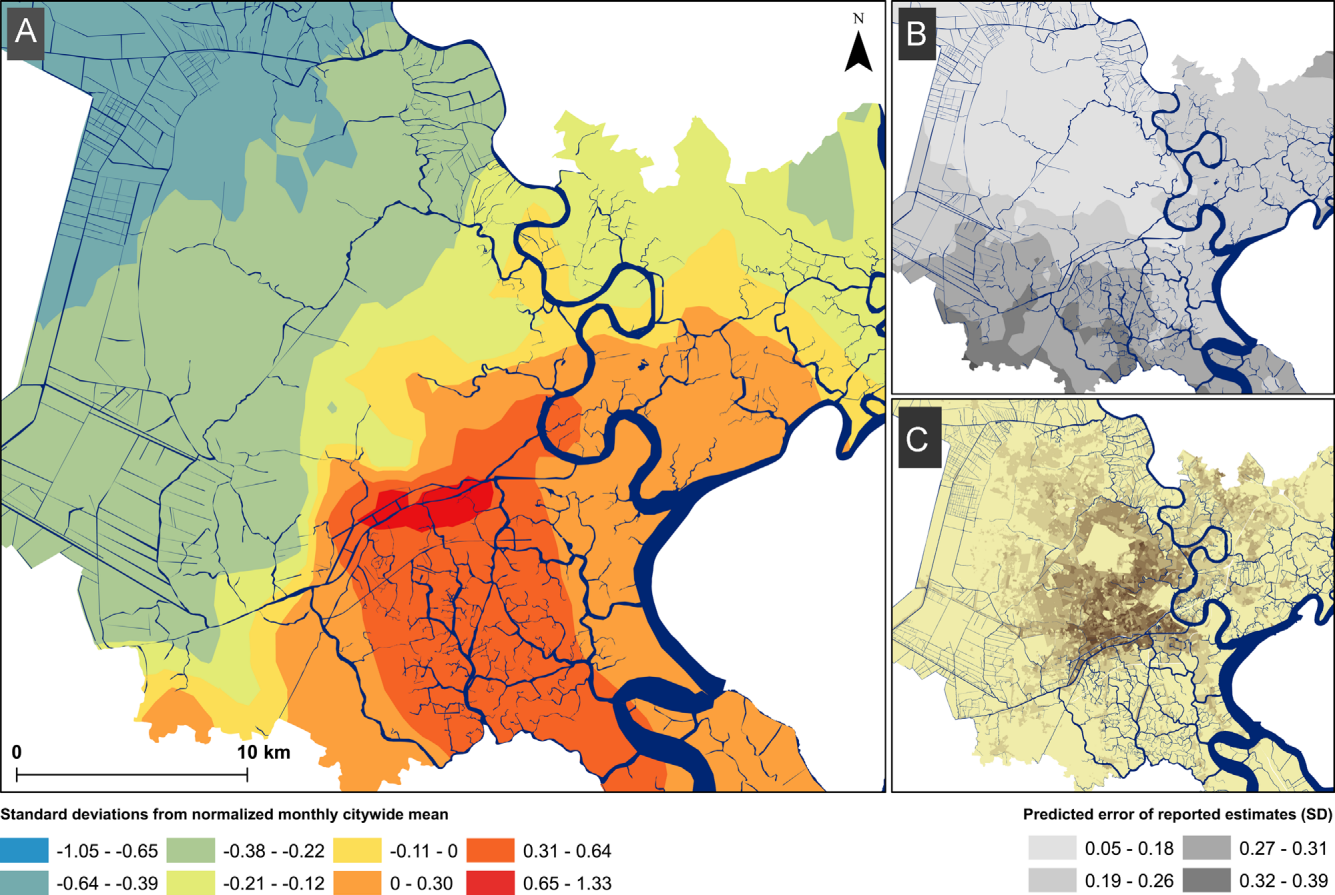
The rate of reported diarrhea in Ho Chi Minh City, 2008–2010. (A) Map of HCMC showing the smoothed rate of reported diarrheal cases per 100,000 population by ward, with the scale in units of standard deviations from normalized monthly citywide mean. Districts are labeled by number in black. (B) Map showing the corresponding population density of HCMC, with darker colors indicating higher densities per square kilometer. (C) Map showing the predicted error of reported diarrheal rate estimates across HCMC, with darker colors indicating increasing uncertainty (scale shown in bottom right of figure, interpreted as standard deviations from predicted local estimate). (For interpretation of the references to color in this figure legend, the reader is referred to the web version of this article.)

The minimum district-level rate of hospitalized diarrhea in children <16 years of age was 11.9/100,000 population (range: 1.1–42.2/100,000). The smoothed ward-level rates in Fig. 2A illustrate substantial heterogeneity in overall risk of diarrheal disease across HCMC. We observed that wards situated adjacent to waterways in the central regions of the city were more likely to have a higher rate of all-cause diarrhea in comparison to the normalized citywide monthly mean. The minimum reported incidence rates in the waterway-laden areas (specifically in wards within district 6 and 8) exceeded 50 cases/100,000 population in some months. These central areas are amongst the most densely populated in the city with a mean population density of 44,300/km^2^ (Fig. 2C), and are situated below the average city elevation (2.8 m) at a ward mean of 1.14 m above sea level (Fig. 2D). The trend of an elevated rate of diarrheal hospitalizations extended south along the river ways and canals through districts 7, 13 and 20 (district locations shown in Fig. 5).

### 3.2 Bivariate analysis

River level, humidity, rainfall and temperature varied seasonally in all study years (Fig. 1B). Using bivariate Poisson regression we found that river level correlated positively with citywide per-capita diarrheal disease rates (Relative Risk (RR):1.07, 95% Confidence Interval (CI):1.06, 1.08), whereas humidity (RR:0.90, 95%CI:0.89, 0.91), rainfall (RR:0.92, 95%CI:0.91, 0.93) and temperature (RR:0.98, 95%CI:0.97, 0.99) were significantly negatively associated with the rate of diarrheal hospitalization throughout 2005–2010.

### 3.3 District-level model

**Fig. 3:**
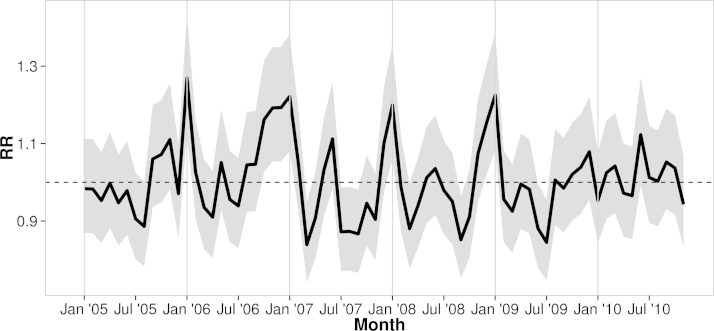
The citywide monthly relative risk of diarrheal disease across Ho Chi Minh City. The solid line in the figure shows the relative risk (RR) of diarrheal disease, as compared to the population-level intercept in Table 1. The shaded region represents the 95% confidence interval for each of the monthly effects. The dashed line is a guide for assessing statistical significance; monthly effects spanning this line are significantly different from the average rate.

**Fig. 4:**
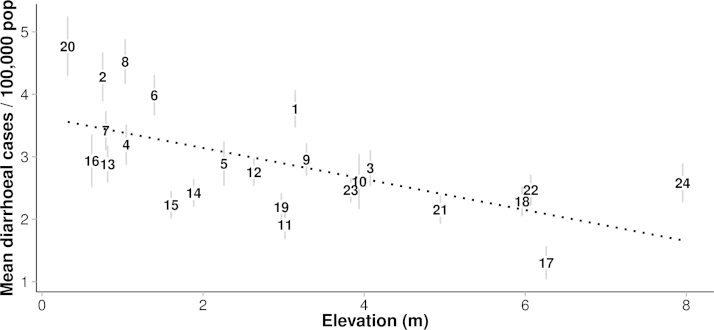
The relationship between the predicted rate of reported diarrheal cases and elevation in Ho Chi Minh City, 2008–2010. Scatterplot showing the estimated average reporting rate by district for 2008–2010 (with bars indicating 95% credible interval) and average district elevation in meters above sea-level. The numbers within the plot represent the point estimate for each of the corresponding districts. The dashed line represents the fitted linear model.

In each year, the relative risk of reported diarrhea at the city level rose rapidly during the final weeks of the year, followed by a peak period lasting from 1 to 2 months. The citywide intercepts for each month of the study are highlighted in Fig. 3. Further, the elevation of each district was strongly related to the reported rate of diarrheal illness (Table 1 and Fig. 4). The RR of a one-meter increase in elevation was 0.95 (95%CI:0.92, 0.97). Therefore, the district with the lowest median elevation (0.32 m) experienced nearly double the risk of the highest district (7.95 m) (RR=0.57, 95%CI:0.31, 0.74). In further models incorporating both elevation and waterway coverage as fixed effects, the influence of waterways was non-significant, although this became significant when elevation was excluded. However, the inverse relationship between average district elevation and the proportion of district area covered by waterways (Pearson's *R*=−0.59, 95%CI:−0.80, −0.25) suggest that waterway coverage may explain some of the risk associated with lower elevation. Finally, district-level elevation was not associated with distance from CH1 (*β*=0.07, 95%CI=−2.76, 2.90), suggesting that adjustment for distance to the largest and most centrally located hospital does not confound the effect of elevation.

**Fig. S4:**
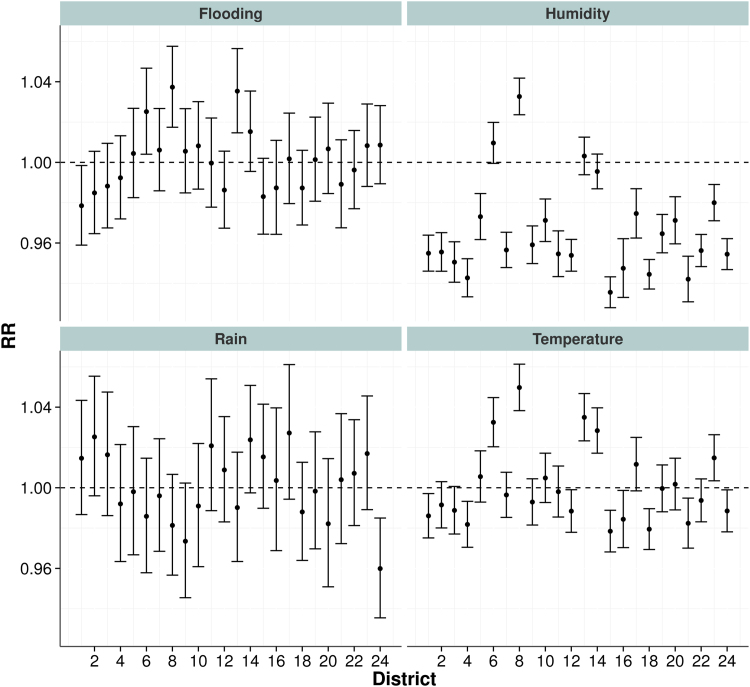


**Fig. S5:**
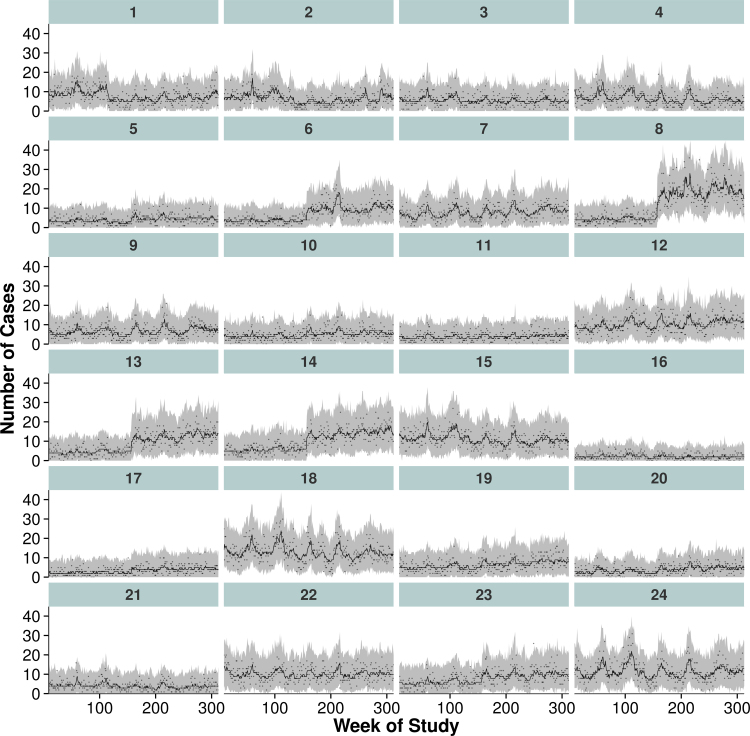


**Fig. S6:**
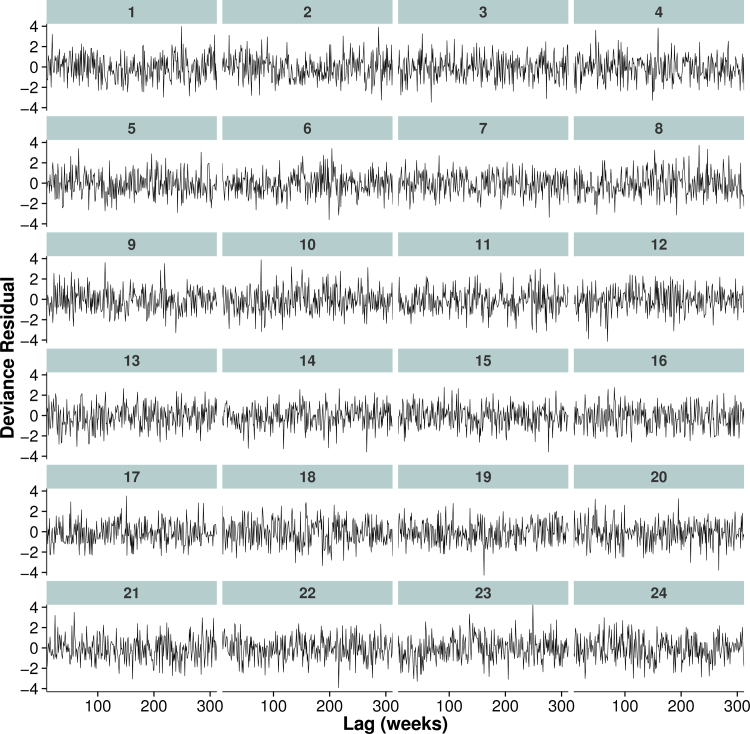


**Fig. S7:**
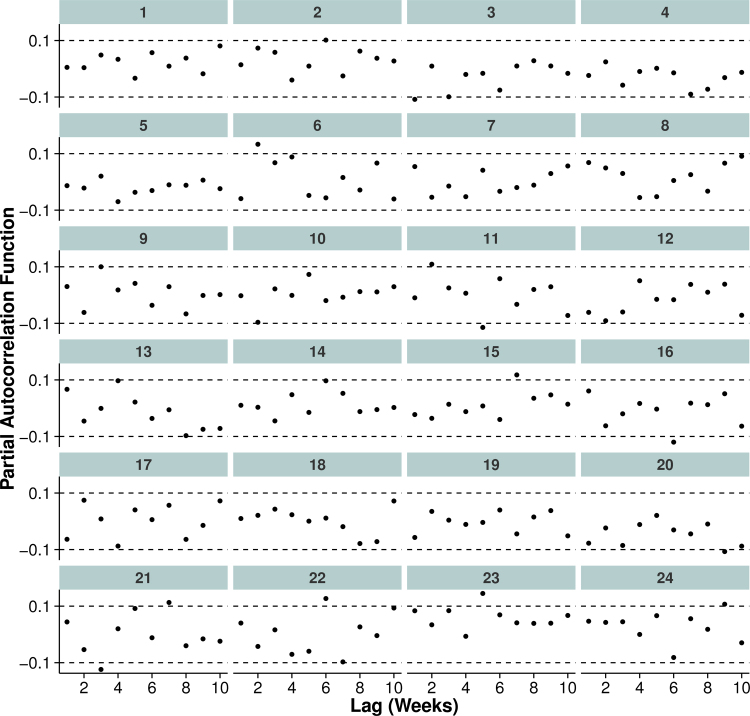


**Fig. 5:**
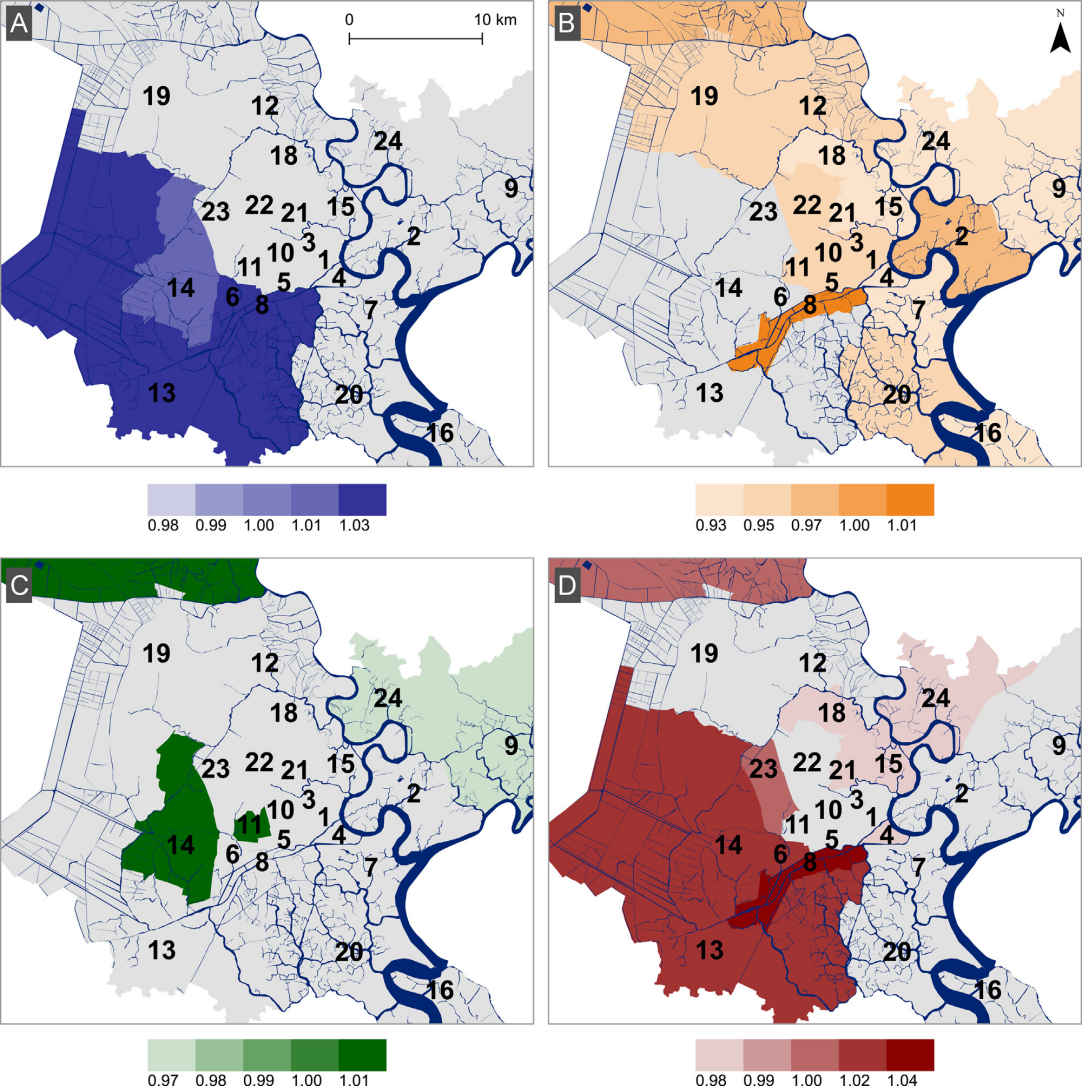
District-level effects of weather and climate variables on diarrheal disease in Ho Chi Minh City. Maps showing the estimated effect scaled to standard deviation of each mean standardized climate variable (A) average weekly river level, (B) humidity, (C) rainfall and (D) temperature across the districts of HCMC. Intensity of color represents the magnitude of estimated coefficients, as indicated by legends. Gray areas indicate non-significant effects. Blue lines in the maps represent rivers and canals. Black numbers are district identifiers. (For interpretation of the references to color in this figure legend, the reader is referred to the web version of this article.)

After accounting for seasonal and district-level effects, heterogeneity in the influence of weather and climate variables at the district level remained (Fig. 5 and Fig. S4). The district-level effect of 1SD increase in flooding on risk of diarrhea was significant for districts 6 (RR:1.02, 95%CI:1.01, 1.04), 8 (RR:1.04, 95%CI:1.02, 1.06) and 13 (RR:1.03, 95%CI:1.02, 1.05); located in the central/southwestern regions of HCMC (Fig. 5A). To understand how these effects impact risk, a change from the minimum observed river level (−2SD) to the maximum (2.3SD) in district 8, for example, correspond to a roughly 20% increase in incidence (RR=1.2, 95%CI:1.1, 1.3), whereas in district 15, the same change is not associated with a significant change in disease risk (RR=0.91, 95%CI:0.8, 1.0) (Table S3). We found that increasing humidity was inversely associated with reported diarrheal disease incidence in most districts of the city, particularly in the northeastern districts (Fig. 5B). District 8 was an exception to this trend, in which there was a positive association between diarrheal hospitalization and humidity (RR:1.03, 95%CI:1.02, 1.05). The citywide average and district-level effect of rainfall was non-significant across most districts (Fig. 5C). Finally, the direction of district-level association between temperature and risk varied across districts. In some districts (6, 8, 13, 14 and 23) this association was positive, whereas in others (districts 1, 4, 12, 13, 16, 18, 21 and 24) the effect appeared to be protective (Fig. 5D and Table S2). A change from the minimum observed temperature (−3.17SD) to maximum (3.09SD) in district 8, for example, would have an RR of 1.37 (95%CI:1.3, 1.5) whereas in district 4 this same change is associated with a slight protective effect (RR =0.84, 95%CI:0.8, 1.0).

## 4 Discussion

Our estimates of how climate and environmental factors impact diarrheal disease risk across HCMC can inform future climate-based early warning systems for diarrhea epidemics. We highlight substantial spatial heterogeneity in patterns of hospitalized diarrhea across districts of HCMC and show that this heterogeneity is driven in part by local variation in the impact of weather and environmental factors. Our multivariate analysis strongly indicates that the increased rates of hospitalization from low-lying areas is a consequence of local environmental conditions in these districts rather than their proximity to the hospitals that provided the data for these analyses. Future climate-based forecasting of diarrheal disease epidemics in other settings should incorporate the localized and heterogeneous nature of risk to allow for efficient targeting of scare resources such as preemptive rotavirus vaccination or provision of water filtration devices (Akanda et al., 2014, Lantagne and Clasen, 2012).

After adjusting for citywide monthly effects, we identified several spatial patterns in the impact of environmental factors on diarrheal disease risk. Southwestern HCMC was more likely to experience an increase in diarrheal hospitalizations during periods with increased river level, relative humidity and temperature. In the northeast of HCMC, however, diarrhea was reported more frequently during periods of low humidity. District 8 was consistently at high risk due to a number of factors including warmer air temperature, flooding, and humidity, making this region particularly vulnerable to predicted effects of climate change. Areas of the city most sensitive to variation in air temperature, such as district 8, may be more likely to experience bacterial or parasitic diarrheal disease, due to the enhanced growth and survival of bacterial and parasitic pathogens in warmer, wetter conditions (Rose et al., 2001).

District elevation was a consistent risk for all-cause diarrhea in this setting. HCMC is low lying, with 40–45% of land lying between 0 and 1 m above sea level (Asian Development Bank, 2010). Because low elevation and surface water coverage are so highly correlated in this setting, it is likely that the effect of low elevation on diarrhea risk is partially facilitated by increased exposure to contaminated surface and ground water (Wust et al., 2002). Flooding events are well documented to increase the risk of childhood diarrhea due to drinking water contamination (Ten Veldhuis et al., 2010, Wu et al., 2013). The canal water in HCMC is known to be heavily contaminated with human and animal waste and heavy metals due to limited restrictions on industrial and domestic waste discharge (Ha et al., 2008, Wust et al., 2002). The poorest residents, who often live in settlements along canals in low-lying districts near central HCMC, normally resort to use of such groundwater through drilled wells (Wust et al., 2002). The strong effect of low elevation, compared to those of local climate, suggests that persistent exposure to contaminated ground water plays a major role in diarrheal risk with effects that are amplified by seasonal variation in climate.

A study by the Asian Development Bank (ADB) highlighted the central districts of 4, 6 and 8 as most at risk of extreme flood events in the future and that this vulnerability may have serious implications for the risk of diarrheal disease and child health in these areas (Asian Development Bank, 2010). Our findings suggest that districts 13, 14 and 23 may be additionally vulnerable to mid- to long-term changes in climate and increasingly volatile weather trends with respect to diarrheal diseases in children. To stymie this growth, future work should include etiological diarrheal surveillance studies to disaggregate pathogen-specific trends to tailor prevention mechanisms more appropriately. Further studies could also use the presented modeling framework to examine the influence of climate and environment on other syndromes recorded routinely at hospital such as pediatric febrile and respiratory infections.

An important limitation of our findings is that our analysis relies on hospitalized diarrhea only. We are, therefore, unable to capture the factors that drive less severe, unreported cases that occur in the community. The trends we report are therefore applicable to our understanding of only the most severe diarrheal cases in the population who are able to attend the selected healthcare facilities. Furthermore, many of our cases had only district of residence recorded in the hospital report so we were unable to perform our analysis on a ward scale. Secondly, our results may be impacted by variation in socioeconomic status (SES) at the household and district level, which we were not able to observe. While measures of average household income exist for HCMC at the city level, reliable data on income and other measures of quality of life at higher resolution, i.e. at the district level, are not available. Recent work highlights how SES factors may impact susceptibility to environmental drivers of risk (Leckebusch and Abdussalam, 2015). In our data, households with low SES may be more likely to live in regions of HCMC with low elevation and poor WASH infrastructure; low SES may thereby drive the true risk of diarrheal disease. The frequency of health seeking behavior may also be correlated with SES and thus regions with lower SES, particularly those in semi-rural areas, may be underrepresented, although this issue is likely mitigated by adjustment for travel distance to central HCMC. However, the use of a random effects model allows us to capture spatial variation in risk that may be due to these unobserved socioeconomic factors. In addition, studies in other low-income, developing contexts with inadequate sanitation have shown that living at low elevation is often associated with low SES, as a consequence of the flooding and disease risk these areas are often subject to (Baker et al., 2011). Nonetheless, the absence of these data highlight the critical need for systematic collection of high-resolution socioeconomic data alongside the types of environmental and hospital and health information presented here.

In conclusion, we identified considerable spatial heterogeneity in patterns of all-cause diarrheal disease in HCMC as well as meaningful district-level variation in sensitivity to climate and environmental factors. We focused on these citywide and localized patterns and drivers of this multi-pathogen syndrome with the aim of informing future climate-based predictive models of diarrheal disease in similar settings. Effective forecasting models of diarrhea in this setting would be a valuable tool when combined with existing or strengthened treatment protocols, breastfeeding promotion, and rotavirus immunization for effective and targeted reduction of diarrheal disease in children. Furthermore, quantification of the effects of weather and environment on risk of pediatric diarrheal disease will become increasingly important for future prevention and control policies, particularly in the face of predicted effects of climate change in both HCMC as well as other similarly high-risk settings (Asian Development Bank, 2010, Patz et al., 2005).

## Conflicts of interest

The authors state that they have no conflicts of interest.

## Ethical approval

Ethical approval for analysis of this anonymized data was granted by all local hospital ethical review committees (Children's Hospital 1, Children's Hospital 2 and the Hospital for Tropical Diseases) as well as the Oxford University Tropical Research Ethics Committee (OxTrec: 1045-13).

## Financial support

This work was supported through the Wellcome Trust Vizions strategic award (WT1093824). SB is a Sir Henry Dale Fellow, jointly funded by the Wellcome Trust and the Royal Society (100087/Z/12/Z). JLZ and BTG were supported by the Research and Policy for Infectious Disease Dynamics program of the Science and Technology Directorate, Department of Homeland Security and the Fogarty International Center, National Institutes of Health. BTG was funded by the Bill and Melinda Gates Foundation. The sponsors of the study had no role in the study design, data collection, data analysis, data interpretation, or writing of the report.

## Figures and Tables

**Table 1:** Fixed effect coefficients from the mixed-effect model.

**Variable**	**RR**	**95% CI**	***p***
Intercept	1.9777	1.608, 2.432	<0.0001
Elevation	0.9478	0.922, 0.974	0.00016
Log(CH1 Distance)	0.9687	0.902, 1.040	0.38026
Lag 1 Week	1.0464	1.039, 1.053	<0.0001
Lag 2 Week	1.0258	1.019, 1.033	<0.0001
Lag 3 Week	1.0134	1.006, 1.020	0.00014
Lag 4 Week	1.0145	1.008, 1.022	<0.0001
Lag 5 Week	1.0071	1.000, 1.014	0.04378
Lag 6 Week	1.0197	1.013, 1.027	<0.0001
Lag 7 Week	1.0095	1.003, 1.016	0.0074
Lag 8 Week	1.0057	0.999, 1.013	0.10213

RR: relative risk; CH1: Children's Hospital 1.

## Footnotes

Appendix A Supplementary data associated with this article can be found in the online version at: doi:10.1016/j.healthplace.2015.08.001
