# Supplementary material for: The impact of environmental and climatic variation on the spatiotemporal trends of hospitalized pediatric diarrhea in Ho Chi Minh City, Vietnam
Source: Health Place. 2015 Sep;35:147–54. doi: 10.1016/j.healthplace.2015.08.001 (PMC4664115; doi:10.1016/j.healthplace.2015.08.001)
Supplement: Supplementary file 1 — Supplementary Material [file mmc1.docx]

**Supporting Information**

**Title:** The impact of environmental and climatic variation on the spatiotemporal trends of hospitalized pediatric diarrhea in Ho Chi Minh City, Vietnam

**Tables**

Supplementary Table 1: District labels used in manuscript and district names of Ho Chi Minh City, Vietnam

| **District Label** | **District Name** |
| --- | --- |
| 1 | 1 |
| 2 | 2 |
| 3 | 3 |
| 4 | 4 |
| 5 | 5 |
| 6 | 6 |
| 7 | 7 |
| 8 | 8 |
| 9 | 9 |
| 10 | 10 |
| 11 | 11 |
| 12 | 12 |
| 13 | Bình Chánh |
| 14 | Bình Tân |
| 15 | Bình Thạnh |
| 16 | Cần Giờ |
| 17 | Củ Chi |
| 18 | Gò Vấp |
| 19 | Hóc Môn |
| 20 | Nhà Bè |
| 21 | Phú Nhuận |
| 22 | Tân Bình |
| 23 | Tân Phú |
| 24 | Thủ Đức |

**Supplementary Table 2:** District level effects of humidity (%), flooding (cm above or below mean), rainfall (mm) and temperature (⁰C), relative risks (RR) and 95% confidence interval (CI) scaled to standard deviation of each mean-standardized climate variable.

| **District** | **Humidity** | | **Flooding** | | **Rain** | | **Temperature** | |
| --- | --- | --- | --- | --- | --- | --- | --- | --- |
|  | RR | 95%CI | RR | 95%CI | RR | 95%CI | RR | 95%CI |
| 1 | 0.972 | 0.955 , 0.989 | 0.985 | 0.967 , 1.002 | 1.007 | 0.985 , 1.029 | 0.992 | 0.981 , 1.004 |
| 2 | 0.975 | 0.957 , 0.993 | 0.991 | 0.973 , 1.009 | 1.019 | 0.997 , 1.043 | 0.999 | 0.987 , 1.011 |
| 3 | 0.960 | 0.941 , 0.979 | 0.992 | 0.974 , 1.011 | 1.012 | 0.988 , 1.036 | 0.993 | 0.981 , 1.006 |
| 4 | 0.938 | 0.921 , 0.956 | 0.990 | 0.972 , 1.008 | 0.998 | 0.976 , 1.021 | 0.982 | 0.970 , 0.995 |
| 5 | 0.974 | 0.954 , 0.994 | 1.004 | 0.984 , 1.023 | 1.001 | 0.977 , 1.026 | 1.008 | 0.994 , 1.021 |
| 6 | 1.012 | 0.993 , 1.030 | 1.024 | 1.006 , 1.043 | 0.989 | 0.967 , 1.012 | 1.035 | 1.022 , 1.048 |
| 7 | 0.952 | 0.935 , 0.968 | 1.006 | 0.988 , 1.024 | 0.997 | 0.976 , 1.019 | 0.997 | 0.985 , 1.009 |
| 8 | 1.032 | 1.016 , 1.049 | 1.038 | 1.020 , 1.056 | 0.982 | 0.962 , 1.001 | 1.051 | 1.039 , 1.063 |
| 9 | 0.946 | 0.929 , 0.964 | 1.003 | 0.985 , 1.022 | 0.976 | 0.955 , 0.999 | 0.991 | 0.979 , 1.003 |
| 10 | 0.966 | 0.947 , 0.986 | 1.006 | 0.987 , 1.025 | 0.996 | 0.972 , 1.020 | 1.005 | 0.992 , 1.019 |
| 11 | 0.956 | 0.936 , 0.976 | 0.998 | 0.979 , 1.018 | 1.026 | 1.000 , 1.051 | 1.001 | 0.987 , 1.014 |
| 12 | 0.960 | 0.945 , 0.976 | 0.989 | 0.972 , 1.006 | 1.006 | 0.986 , 1.027 | 0.992 | 0.981 , 1.003 |
| 13 | 0.993 | 0.976 , 1.010 | 1.034 | 1.015 , 1.052 | 0.993 | 0.972 , 1.014 | 1.034 | 1.022 , 1.047 |
| 14 | 1.004 | 0.988 , 1.021 | 1.018 | 1.001 , 1.036 | 1.021 | 1.000 , 1.042 | 1.033 | 1.021 , 1.045 |
| 15 | 0.935 | 0.920 , 0.949 | 0.983 | 0.967 , 1.000 | 1.015 | 0.995 , 1.036 | 0.980 | 0.969 , 0.991 |
| 16 | 0.950 | 0.927 , 0.974 | 0.988 | 0.968 , 1.008 | 1.005 | 0.977 , 1.033 | 0.987 | 0.972 , 1.002 |
| 17 | 0.978 | 0.957 , 0.999 | 1.003 | 0.984 , 1.023 | 1.027 | 1.001 , 1.053 | 1.015 | 1.001 , 1.029 |
| 18 | 0.941 | 0.927 , 0.955 | 0.987 | 0.970 , 1.004 | 0.989 | 0.970 , 1.008 | 0.980 | 0.969 , 0.991 |
| 19 | 0.960 | 0.942 , 0.978 | 1.000 | 0.982 , 1.019 | 1.000 | 0.978 , 1.023 | 1.000 | 0.988 , 1.013 |
| 20 | 0.970 | 0.950 , 0.991 | 1.006 | 0.987 , 1.025 | 0.985 | 0.961 , 1.010 | 1.003 | 0.990 , 1.017 |
| 21 | 0.941 | 0.921 , 0.961 | 0.989 | 0.970 , 1.008 | 1.005 | 0.980 , 1.030 | 0.984 | 0.971 , 0.997 |
| 22 | 0.964 | 0.949 , 0.980 | 0.998 | 0.981 , 1.016 | 1.006 | 0.986 , 1.027 | 0.998 | 0.986 , 1.009 |
| 23 | 0.987 | 0.969 , 1.004 | 1.012 | 0.994 , 1.030 | 1.012 | 0.990 , 1.034 | 1.019 | 1.007 , 1.031 |
| 24 | 0.942 | 0.927 , 0.957 | 1.004 | 0.987 , 1.022 | 0.967 | 0.947 , 0.986 | 0.986 | 0.976 , 0.998 |

**Supplementary Table 3:** District level relative risks (RR) and 95% confidence intervals (CI) for a change from the minimum level of each climate variable to the maximum level

| **District** | **Humidity** | | **Flooding** | | **Rainfall** | | **Temperature** | |
| --- | --- | --- | --- | --- | --- | --- | --- | --- |
|  | RR | 95%CI | RR | 95%CI | RR | 95%CI | RR | 95%CI |
| 1 | 0.833 | 0.732, 0.935 | 0.921 | 0.827, 1.010 | 1.044 | 0.905, 1.183 | 0.942 | 0.862, 1.029 |
| 2 | 0.851 | 0.744, 0.958 | 0.953 | 0.859, 1.047 | 1.120 | 0.981, 1.272 | 0.993 | 0.906, 1.080 |
| 3 | 0.762 | 0.649, 0.875 | 0.958 | 0.864, 1.058 | 1.076 | 0.924, 1.228 | 0.949 | 0.862, 1.044 |
| 4 | 0.631 | 0.530, 0.738 | 0.948 | 0.853, 1.042 | 0.987 | 0.848, 1.133 | 0.869 | 0.782, 0.964 |
| 5 | 0.845 | 0.726, 0.964 | 1.021 | 0.916, 1.120 | 1.006 | 0.855, 1.164 | 1.058 | 0.956, 1.152 |
| 6 | 1.071 | 0.958, 1.178 | 1.126 | 1.031, 1.225 | 0.930 | 0.791, 1.076 | 1.254 | 1.160, 1.349 |
| 7 | 0.715 | 0.613, 0.810 | 1.031 | 0.937, 1.126 | 0.981 | 0.848, 1.120 | 0.978 | 0.891, 1.065 |
| 8 | 1.190 | 1.095, 1.291 | 1.199 | 1.105, 1.293 | 0.886 | 0.760, 1.006 | 1.370 | 1.283, 1.457 |
| 9 | 0.679 | 0.578, 0.786 | 1.016 | 0.921, 1.115 | 0.848 | 0.716, 0.994 | 0.935 | 0.848, 1.022 |
| 10 | 0.798 | 0.685, 0.917 | 1.031 | 0.932, 1.131 | 0.975 | 0.823, 1.126 | 1.036 | 0.942, 1.138 |
| 11 | 0.738 | 0.619, 0.857 | 0.990 | 0.890, 1.094 | 1.164 | 1.000, 1.322 | 1.007 | 0.906, 1.102 |
| 12 | 0.762 | 0.673, 0.857 | 0.942 | 0.853, 1.031 | 1.038 | 0.912, 1.171 | 0.942 | 0.862, 1.022 |
| 13 | 0.958 | 0.857, 1.059 | 1.178 | 1.079, 1.272 | 0.956 | 0.823, 1.088 | 1.247 | 1.160, 1.341 |
| 14 | 1.024 | 0.929, 1.125 | 1.094 | 1.005, 1.189 | 1.133 | 1.000, 1.265 | 1.240 | 1.152, 1.327 |
| 15 | 0.613 | 0.524, 0.697 | 0.911 | 0.827, 1.000 | 1.095 | 0.968, 1.228 | 0.855 | 0.775, 0.935 |
| 16 | 0.703 | 0.566, 0.845 | 0.937 | 0.832, 1.042 | 1.032 | 0.855, 1.209 | 0.906 | 0.797, 1.015 |
| 17 | 0.869 | 0.744, 0.994 | 1.016 | 0.916, 1.120 | 1.171 | 1.006, 1.335 | 1.109 | 1.007, 1.211 |
| 18 | 0.649 | 0.566, 0.732 | 0.932 | 0.843, 1.021 | 0.930 | 0.810, 1.051 | 0.855 | 0.775, 0.935 |
| 19 | 0.762 | 0.655, 0.869 | 1.000 | 0.906, 1.100 | 1.000 | 0.861, 1.145 | 1.000 | 0.913, 1.094 |
| 20 | 0.822 | 0.703, 0.946 | 1.031 | 0.932, 1.131 | 0.905 | 0.754, 1.063 | 1.022 | 0.927, 1.123 |
| 21 | 0.649 | 0.530, 0.768 | 0.942 | 0.843, 1.042 | 1.032 | 0.874, 1.190 | 0.884 | 0.789, 0.978 |
| 22 | 0.786 | 0.697, 0.881 | 0.990 | 0.900, 1.084 | 1.038 | 0.912, 1.171 | 0.985 | 0.898, 1.065 |
| 23 | 0.923 | 0.816, 1.024 | 1.063 | 0.969, 1.157 | 1.076 | 0.937, 1.215 | 1.138 | 1.051, 1.225 |
| 24 | 0.655 | 0.566, 0.744 | 1.021 | 0.932, 1.115 | 0.791 | 0.665, 0.912 | 0.898 | 0.826, 0.985 |

Minimum and maximum mean-normalized values (1) humidity: -2.7SD, 2.2SD (2) flooding: -2.0, 2.3 (3) rainfall: -0.9, 4.5 (4) temperature: -3.2, 3.1

**Supplementary Figure Legends**

**Supplementary Figure 1**: **Population growth by district over time**. To account for changing population size over the period included in the analysis (2005-2010), we estimated weekly district-level population sizes using linear interpolation, assuming a constant rate of change in district population size between available census observations from 2005, 2008, 2009 and 2010 (*Statistical Yearbook of Ho Chi Minh City 2011*, 2012).

**Supplementary Figure 2: District level random intercept and change in reporting from 2008-2010.** (A) District level variability in baseline risk assumed to apply consistently over time, shown in relative risk in terms of deviation from overall average rate. (B) District level relative risk of reporting in any period after 2008, again shown in terms of deviation from overall average rate. Dashed lines in each panel are provided as a guide for assessing statistical significance.

**Supplementary Figure 3: District level time series**. Count of diarrheal cases per week over the study period (2005-2010) by district.

**Supplementary Figure 4: District level weather and climate effects.** Relative risks of flooding, humidity, rainfall and temperature by district predicted from the mixed effects model. The dashed line in each panel is provided as a guide for assessing statistical significance.

**Supplementary Figure 5**: **Goodness of fit by district.** Each panel illustrates the fit of the model to the data from each district using 1000 simulations from the fitted model. Points indicate the number of cases for each week. The solid line shows the median simulated value for each week, and the gray shaded area shows the range from the minimum to maximum simulated values.

**Supplementary Figure 6: District level deviance residuals.** Each panel of the plot shows the deviance residuals for each district after accounting for fixed effects, and seasonal and district-level random effects.

**Supplementary Figure 7: Partial autocorrelation function.** Partial autocorrelation function (PACF) of district-level residuals. Points in each panel of the plot show the PACF for each district at each week of a 10-week lag. Dashed lines at 0.1 and -0.1 show a range of values indicating minimal residual temporal autocorrelation.

**References**

Statistical Yearbook of Ho Chi Minh City 2011, 2012. . Ho Chi Minh City Statistical Office, Ho Chi Minh City.
